# Emerging Importance of Chemokine Receptor CXCR4 and Its Ligand in Liver Disease

**DOI:** 10.3389/fcell.2021.716842

**Published:** 2021-07-27

**Authors:** Sheng Wang, Songsen Gao, Yueran Li, Xueyi Qian, Jiajie Luan, Xiongwen Lv

**Affiliations:** ^1^Department of Pharmacy, The First Affiliated Hospital of Wannan Medical College, Yijishan Hospital of Wannan Medical College, Wuhu, China; ^2^The Key Laboratory of Anti-inflammatory and Immune Medicines, Ministry of Education, School of Pharmacy, Institute for Liver Disease of Anhui Medical University, Hefei, China; ^3^Department of Orthopedics (Spinal Surgery), The First Affiliated Hospital of Anhui Medical University, Hefei, China

**Keywords:** CXCR4, CXCL12, liver specific cells, stem cells, targeted therapy, liver disease

## Abstract

Chemokine receptors are members of the G protein-coupled receptor superfamily, which together with chemokine ligands form chemokine networks to regulate various cellular functions, immune and physiological processes. These receptors are closely related to cell movement and thus play a vital role in several physiological and pathological processes that require regulation of cell migration. CXCR4, one of the most intensively studied chemokine receptors, is involved in many functions in addition to immune cells recruitment and plays a pivotal role in the pathogenesis of liver disease. Aberrant CXCR4 expression pattern is related to the migration and movement of liver specific cells in liver disease through its cross-talk with a variety of significant cell signaling pathways. An in-depth understanding of CXCR4-mediated signaling pathway and its role in liver disease is critical to identifying potential therapeutic strategies. Current therapeutic strategies for liver disease mainly focus on regulating the key functions of specific cells in the liver, in which the CXCR4 pathway plays a crucial role. Multiple challenges remain to be overcome in order to more effectively target CXCR4 pathway and identify novel combination therapies with existing strategies. This review emphasizes the role of CXCR4 and its important cell signaling pathways in the pathogenesis of liver disease and summarizes the targeted therapeutic studies conducted to date.

## Introduction

Liver disease is a leading cause of illness and death in the world (Wang et al., 2014; Marcellin and Kutala, 2018). In recent years, the incidence of liver disease such as alcoholic liver disease (ALD), non-alcoholic fatty liver disease (NAFLD), viral hepatitis, liver fibrosis and cirrhosis, hepatocellular carcinoma (HCC) and liver failure (LF) has gradually increased (Wang et al., 2014, 2021). Because the molecular mechanism of liver disease is very complicated, there is still no clinically effective treatment for specific pathogenesis. The current academic opinion holds that specific cells in the liver play a significant role in the pathophysiology of liver disease (Poisson et al., 2017; Cai B. et al., 2020). However, how these cells play a role in liver disease, and the specific molecular mechanisms that regulate cellular functions are still not fully elucidated. Therefore, in-depth study of liver disease progression mechanisms and specific cellular functions, as well as the determination of crucial node molecules are important scientific problems to be solved in the field of liver disease research.

**FIGURE 1:**
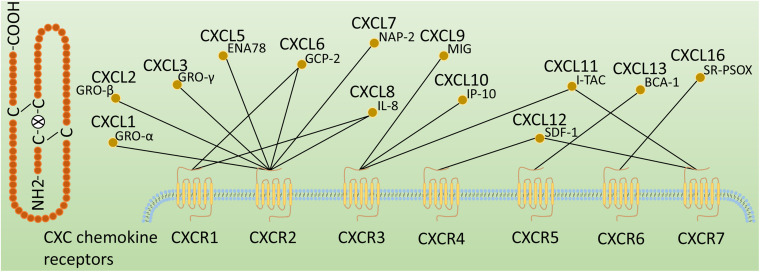
Classification of CXC chemokine receptors. CXC chemokine receptors are classified according to the ligands they bind, followed by an R (representing receptor) and a number corresponding to the order of discovery. These CXC chemokine ligands are also known as GRO-α (CXCL1), GRO-β (CXCL2), GRO-γ (CXCL3), ENA78 (CXCL5), GCP-2 (CXCL6), NAP-2 (CXCL7), IL-8 (CXCL8), MIG (CXCL9), IP-10 (CXCL10), I-TAC(CXCL11), SDF-1(CXCL12), BCA-1(CXCL13), and SR-PSOX(CXCL16).

Chemokines, also known as chemotactic cytokines, are a large family of small and secreted proteins with molecular weights in the range of 8–12 kDa that are involved in a variety of cellular functions, including inducing target cell chemotaxis (migration), guiding cell movement and mediating immune cell trafficking (Roussos et al., 2011; Kolaczkowska and Kubes, 2013; Griffith et al., 2014; Chen w. et al., 2018; Laufer et al., 2019). Chemokines have become a large family of more than 50 members. Chemokine receptors are 7-transmembrane (7TM) G protein-coupled receptors (GPCRs), which are subdivided into four types (CXCR, CX3CR, XCR, and CCR) according to the class of chemokines they bind (Bussmann and Raz, 2015). Among them, CXC chemokine receptors, including CXCR1 to CXCR7, are one of the largest chemokine families and play important roles in several physiological and pathological processes (Ullah, 2019). They are mainly expressed on immune and inflammatory cells and are also present in non-immune cells such as resident cells within the liver (Zlotnik et al., 2011; Choi et al., 2016). CXC chemokine receptors can be grossly defined as inflammatory, homeostatic or dual-function receptors based on the ligands they bind (Zlotnik and Yoshie, 2012). The major ligands of CXC chemokine receptors are shown in Figure 1.

C-X-C motif chemokine receptor 4 (CXCR4) is a chemokine receptor that has been intensively studied because of its significant role in cellular functions, immune processes, growth and development, and liver disease (Qin L. et al., 2018; Zheng et al., 2018; Ullah, 2019; Yang et al., 2019). Its role in liver disease may involve the regulation of the migration and movement of hepatocytes, hepatic stellate cells (HSCs), Kupffer cells (KCs), fibroblasts, endothelial cells and circulating immune cells (Chen w. et al., 2018). In the liver environment, CXCR4 is ubiquitously expressed in most liver cells such as HSCs, KCs and liver sinusoidal endothelial cells (LSECs), but also in malignant cells (Hong et al., 2009; Ding et al., 2013; Wang et al., 2018, 2020). CXCR4 can bind to C-X-C motif chemokine 12 (CXCL12), and CXCL12, also known as stromal cell-derived factor-1 (SDF-1), is the only specific endogenous ligand for CXCR4 (Ullah, 2019). CXCL12 (SDF-1) plays an important role in several physiological and pathological processes by binding to CXCR4 and then participating in cell localization, chemotaxis, activation, migration, proliferation and differentiation (Zhuo et al., 2012; Janssens et al., 2018; Daniel et al., 2020). There is mounting evidence that the CXCR4 and its ligand provide potential targets for the treatment of liver disease, tumors, and cardiovascular disease (Zhuo et al., 2012; Döring et al., 2017; Wang et al., 2018). To date, a number of therapeutic studies have been conducted in combination targeting CXCR4 and its ligand (Sung et al., 2018; Zheng et al., 2019). This review aims to provide a comprehensive overview of the role of CXCR4 and its ligand in liver disease, including its potential as a therapeutic target, and summarize the therapeutic studies of combined targeting CXCR4 pathway.

## Biology of CXCR4 and Its Ligand

Chemokines (chemotactic cytokines) are a family of small and highly conserved proteins that bind to and signal through cell surface 7TM G protein-coupled receptors, which in turn regulate cell migration and function (Hughes and Nibbs, 2018). The four types of chemokine receptors, based on their expression status and functions executed in healthy and disease states, are further divided into constitutively expressed homeostatic chemokine receptors, inducibly expressed inflammatory chemokine receptors, and dual-type chemokine receptors with both characteristics (Mamazhakypov et al., 2021). In addition to the above four types (CXCR, CX3CR, XCR, and CCR), chemokine receptors also include a group of atypical chemokine receptors (ACKRs), which bind to different families of chemokines, but mainly act as decoy and scavenger receptors (Meyrath et al., 2020). Among these chemokine receptors, CXCR1-7, as important members, have been extensively studied in various organs and systems, both in physiological and pathological conditions. For instance, CXCR1 and CXCR2 are involved in the pathogenesis of inflammation and fibrosis (Kormann et al., 2012; Mattos et al., 2020); CXCR3 plays important roles in angiogenesis and tumors (Quemener et al., 2016); CXCR4 regulates the development of hematopoietic and nervous systems, and modulates different cellular functions, including cell migration, chemotaxis, differentiation, growth, activation, proliferation, survival and apoptosis (Murphy and Heusinkveld, 2018); CXCR5 is closely related to immunomodulation (Zhang et al., 2017); CXCR6 and CXCR7 are mainly involved in the regulation of inflammation and cellular functions (Humpert et al., 2014; Butcher et al., 2016; Chang et al., 2018). Interestingly, CXCL12 can also bind to CXCR7 (also known as atypical chemokine receptor 3, or ACKR3), even with a greater affinity than to CXCR4 (Guo et al., 2015; Liepelt and Tacke, 2016). Notably, among CXCR1-7, CXCR4 is the most intensively studied, and it plays an important role in many pathophysiological processes through different signaling pathways.

**FIGURE 2:**
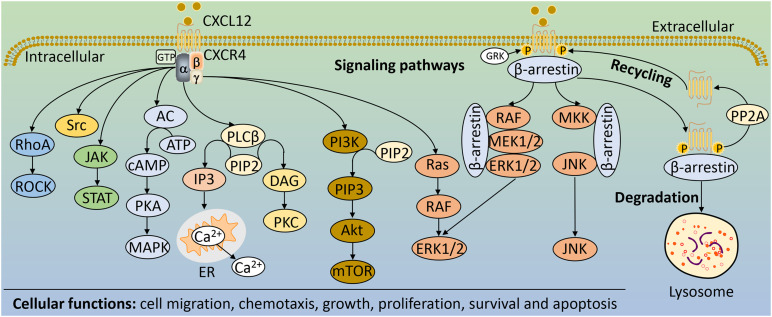
The main signaling pathways and cellular functions of CXCR4. Upon CXCR4 activation, Gα and Gβγ subunits activate different downstream signaling pathways including RhoA/ROCK, cAMP/PKA, PI3K/Akt/mTOR, JAK/STAT, Ras/RAF/ERK1/2, Src, and PLCβ. GRK phosphorylates CXCR4, enabling β-arrestin to bind and internalize CXCR4, which can lead to receptor recycling if receptor phosphorylation is reversed by PP2A or degraded in lysosomes. β-arrestin bound to CXCR4 can also activate MAPK pathways, such as JNK and ERK1/2. These downstream signaling pathways of CXCR4 regulate a variety of cellular functions, such as cell migration, chemotaxis, growth, proliferation, survival and apoptosis. AC, adenylate cyclase; Akt, protein kinase B; ATP, adenosine-5′-triphosphate; cAMP, cyclic adenosine monophosphate; DAG, diacylglycerol; ER, endoplasmic reticulum; IP3, inositol-1,4,5-trisphosphate; ERK1/2, extracellular signal-regulated kinases 1/2; GRK, G protein receptor kinases; JAK, Janus kinase; JNK, c-Jun N-terminal kinase; MAPK, mitogen-activated protein kinase; mTOR, mammalian target of rapamycin; PI3K, phosphoinositide-3-kinase; PIP2, phosphatidylinositol-4,5,-bisphosphate; PIP3, phosphatidylinositol (3,4,5)-trisphosphate; PLCβ, phospholipase Cβ; PKA, protein kinases A; PKC, protein kinases C; PP2A, protein phosphatase 2; RhoA, Ras homolog gene family member A; ROCK, Rho-related PK; STAT, signal transducer and activator of transcription.

Chemokine receptors typically interact with a variety of chemokines to signal, but CXCR4 is an exception, and is specific for the chemokine CXCL12. Together, they constitute CXCR4 pathway that normally play a significant role in the development of multiple systems, but they are also important in disease. CXCR4 signaling is mainly mediated by proteins that interact with receptors, including heterotrimeric G proteins, G protein receptor kinases (GRKs) and β-arrestin adapter proteins (Wang and Knaut, 2014). CXCL12 binding to CXCR7 usually leads to β-arrestin mediated signaling (Daniel et al., 2020). Heterotrimeric G proteins are composed of Gα, Gβ, and Gγ subunits. In the inactive or basal state, the Gα subunit contains guanine nucleotide diphosphate (GDP) (Cojoc et al., 2013). When chemokines stimulate the activation of the receptor CXCR4 and promote interaction between the receptor and the trimeric G-protein α, βγ. This leads to the exchange of GDP for GTP bound to Gα subunits and the dissociation of the Gβγ heterodimers (Mamazhakypov et al., 2021). The dissociated subunits promote downstream signaling through different pathways (Figure 2).

The different pathways of GPCRs signaling depend on the coupled Gα subunits, which are divided into four families: Gαs, Gαi, Gαq, and Gα12. Indeed, CXCR4 can signal by coupling to different families of Gα subunits (Wu et al., 2012; Bar-Shavit et al., 2016). CXCR4 coupled to Gαs stimulates adenylate cyclase (AC), whereas CXCR4 bound to Gαi inhibits AC (Rosciglione et al., 2014; Piovan et al., 2018). AC serves as an effector enzyme that catalyzes the conversion of adenosine-5′-triphosphate (ATP) into cyclic adenosine monophosphate (cAMP), thereby activating cAMP-dependent protein kinase (PKA), which further regulates mitogen-activated protein kinase (MAPK) signaling pathway (Wang et al., 2015; Teixidó et al., 2018). In addition, Gαs and Gαi can also change the activity of Src tyrosine kinase, thereby affecting its role in signal integration (Chiou and Zennadi, 2015). Gαq coupled to CXCR4 converts phosphatidylinositol-4,5,-bisphosphate (PIP2) into inositol-1,4,5-trisphosphate (IP3) and diacylglycerol (DAG) by activating phospholipase Cβ (PLCβ). DAG activates a family of protein kinases (PKs), including PKC, which phosphorylates a number of downstream effectors, such as the Ras signaling pathway (Irnaten et al., 2020); while IP3 diffuses to the endoplasmic reticulum (ER) membrane and binds to gated calcium ion (Ca2^+^) channels, triggering the release of calcium from intracellular storage into the cytoplasm (Dessein et al., 2010). CXCR4 signaling often requires this intracellular calcium mobilization to drive (Engevik et al., 2019). Moreover, CXCR4 coupled to Gα12 further activates Rho-related PK (ROCK) by activating the Ras homolog gene family member A (RhoA), which in turn participates in different cellular functions (Zainal et al., 2018; Figure 2). Activation of phosphoinositide-3-kinase (PI3K) by CXCR4 is predominantly mediated by Gβγ subunits (Teicher and Fricker, 2010). PI3K converts PIP2 to phosphatidylinositol (3,4,5)-trisphosphate (PIP3), triggering a signaling cascade that leads to the activation of the serine/threonine kinase Akt (Akt) and several of its downstream targets, including mammalian target of rapamycin (mTOR) (Qian et al., 2009; Xu et al., 2020). Functionally, in response to CXCR4-mediated signaling, the PI3K/Akt pathway could regulate a variety of cellular activities, including cell migration and survival (Qian et al., 2009; Figure 2).

**FIGURE 3:**
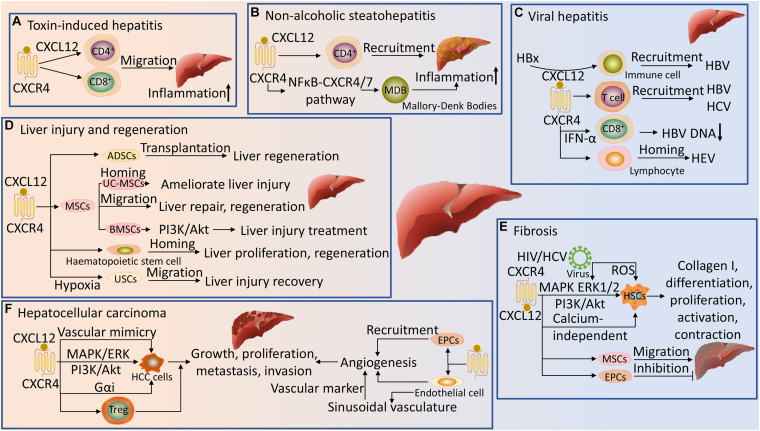
Regulation of liver disease by CXCR4 and its ligand. **(A)** In toxin-induced hepatitis, the CXCL12/CXCR4 axis promotes the migration of CD4^+^ and CD8^+^ cells to the liver and induces aggregation of inflammation. **(B)** CXCL12 is dependent on CXCR4 to promote the recruitment of CD4^+^ T cells in NASH, and the NFκB-CXCR4/7 pathway further promotes inflammation in NASH by forming Mallory-Denk Bodies (MDB). **(C)** The CXCL12/CXCR4 pathway is involved in the recruitment and homing of immune cells in the liver during viral hepatitis (HBV, HCV, and HEV) infection. In HBV, hepatitis B virus X protein (HBx) facilitates recruitment via CXCL12 signaling, and CXCR4 is highly expressed in CD8^+^ T cells after PEGylated interferon-α (INF-α) treatment and inversely correlates with HBV DNA loads. **(D)** The CXCL12/CXCR4 axis promotes migration and homing of mesenchymal stem cells (MSCs), bone marrow mesenchymal stromal stem cells (BMSCs), umbilical cord-derived mesenchymal stem cells (UC-MSCs), adipose-derived stem cells (ADSCs), urine-derived stem cells (USCs) and hematopoietic stem cells to the injured liver to ameliorate liver injury and promote liver repair, proliferation and regeneration. Activation of the PI3K/Akt signaling pathway by CXCR4 in BMSCs could promote cell migration, resulting in better therapeutic effects for liver injury. Hypoxia preconditioning promotes the proliferation and migration of USCs by inducing CXCR4 signaling, leading to recovery from liver injury. **(E)** CXCL12 activation of CXCR4 promotes hepatic stellate cells (HSCs) differentiation, proliferation, activation and contraction via the MAPK, ERK1/2, PI3K/Akt and calcium-independent pathways, inducing collagen I production with fibrotic effects. CXCR4 HIV promotes liver fibrosis by promoting the phosphorylation of ERK1/2 on activated HSCs and inducing reactive oxygen species (ROS) production in HSCs. The CXCL12/CXCR4 axis induces migration of MSCs and endothelial progenitor cells (EPCs) into fibrotic liver, aggravating and inhibiting liver fibrosis, respectively. **(F)** The CXCL12/CXCR4 axis promotes HCC cells growth, proliferation, metastasis and invasion, and vascular mimicry formation via activation of heterotrimeric G proteins, MAPK/ERK and PI3K/Akt signaling pathways; and recruits Treg cells to the tumor sites to promote HCC. The CXCL12/CXCR4 axis enhances the recruitment of endothelial cells and EPCs to HCC and promotes tumor neovascularization. CXCR4 is highly expressed on endothelial cells and can be used as a novel vascular marker for vessel sprouting in HCC tissues.

In addition to signaling via G proteins, activated receptors recruit β-arrestin, which can result in G protein-independent activation of MAPK signaling pathway (Wang and Knaut, 2014). Upon receptor activation, CXCR4 promotes the recruitment of GRKs and other kinases that induce site-specific phosphorylation of the cytoplasmic loops and C-terminus, leading to association with β-arrestin (Pozzobon et al., 2016). β-arrestin mediates receptor desensitization, and targets the receptor for lysosomal degradation following protein internalization and trafficking (Cojoc et al., 2013; Smith and Rajagopal, 2016). Furthermore, β-arrestin also serves as scaffolds for the activation of a variety of signaling molecules, including transcription factors and kinases, such as extracellular signal-regulated kinases 1/2 (ERK1/2) in complex with RAF and MEK1/2 (Figure 2; Mamazhakypov et al., 2021). These pathways, together with the heterotrimeric G proteins-mediated signaling, play important roles in the pathophysiology of diseases, including liver disease (Figure 3).

## The Regulatory Role of CXCR4 and Its Ligand in Hepatitis

Hepatitis is an inflammation of the liver that can be caused by different types of infectious agents such as toxins or viruses (Wang et al., 2021). If left untreated, hepatitis can lead to serious health problems, including liver damage, liver fibrosis and cirrhosis, liver failure, liver cancer, and even death. Although the pathophysiology of hepatitis has not been fully elucidated, many studies have demonstrated the role of CXCR4 and its ligand in hepatitis. CXCR4 normally interacts with CXCL12 to initiate downstream signaling pathways. CXCR4 then plays a crucial role in regulating signal transduction, maintaining the homeostasis of inflammatory responses, and chemotaxis of inflammatory cells. Importantly, the mechanisms of CXCR4 signaling mediated inflammatory responses may affect the effective chemotactic function of inflammatory cells, such as lymphocytes, neutrophils and monocytes (Tian et al., 2019). These inflammatory cells are chemotactic to the site of inflammation and migrate into the tissues, which in turn participates in the inflammatory response of the tissues (Shi and Pamer, 2011). Indeed, in inflammatory liver disease, most liver-infiltrating lymphocytes express CXCR4, and its intensity is more significantly up-regulated in liver-infiltrating lymphocytes than in peripheral blood lymphocytes (Terada et al., 2003). Here, in Concanavalin A-induced T cell mediated hepatitis, the transmigration of CXCR4^+^ total CD4^+^ T cells are enhanced and accumulates in the inflamed liver tissue. This hepatic recruitment of CD4^+^ T cells population is mainly facilitated by LSECs providing perivascularly expressed CXCL12 through CXCR4 dependent intracellular transport mechanisms (Lutter et al., 2015). Consistently, in hepatitis with alcoholic liver disease, CXCR4 dependent migration of lymphocytes into the tissue is significantly increased in response to treatment with ethanol, resulting in recruitment of CD4^+^ and CD8^+^ lymphocytes into liver tissue (Karim et al., 2013). The homing and migration of inflammatory cells to the liver is also critical for the progression of non-alcoholic steatohepatitis (NASH). The pathophysiology of NASH has not been completely elucidated, but it is generally accepted in academia that immune cell recruitment is a crucial factor in initiating and expanding liver inflammation, which contributes to the progression from simple steatosis to NASH (Yu et al., 2019). Here, CXCL12 and CXCR4 protein levels are significantly increased, and CD4^+^ T cells are hyperresponsive to CXCL12 in NASH liver (Bigorgne et al., 2008; Li et al., 2020). Importantly, CXCL12 promotes the recruitment of CD4^+^ T cells in NASH and is dependent on CXCR4, which is attributed to the increased affinity of CXCL12 to CXCR4 (Boujedidi et al., 2014). Moreover, in NASH, Mallory-Denk Bodies (MDB) is formed via the NFκB-CXCR4/7 (CXCR4 and CXCR7) pathway, which in turn participates in ongoing inflammation (Liu H. et al., 2014). Notably, AMD3100, as a CXCR4 antagonist inhibits the chemotactic effect of CXCL12 to CD4^+^ T cells and reduces the number of CD4^+^ T cells that reach the liver (Boujedidi et al., 2014). Thus, CXCR4 and its ligand offer potential targets for pharmacologic therapies for NASH.

The CXCR4 signaling pathway also plays a vital role in virus-induced hepatitis. Viral hepatitis is one of the most common chronic liver disease, and persistent viral infection could lead to liver fibrosis and cirrhosis, HCC and liver failure (Ringehan et al., 2017). One of the important reasons for the progression of viral hepatitis is immune imbalance, which may be mediated by inflammatory cells. Notably, during chronic hepatitis virus infection, chemokine-chemokine receptor interactions are particularly critical for recruiting T cells to sites of inflammation in the liver (Nishitsuji et al., 2013). Indeed, the CXCL12/CXCR4 pathway plays a crucial role in the recruitment and retention of T cells in the liver during chronic hepatitis C virus (HCV) and hepatitis B virus (HBV) infection (Wald et al., 2004). The expression of CXCR4 is significantly enhanced in HCV and HBV-associated hepatitis tissues compared to normal liver tissues (Hong et al., 2009; Boissonnas et al., 2016; Zhu et al., 2016). Notably, in isolated peripheral blood cells from HBV patients treated with PEGylated interferon-α (IFN-α), CXCR4 is also highly expressed in CD8^+^ T cells, which is inversely correlated with HBV DNA loads (Liu et al., 2012). Interestingly, recent studies have shown that hepatitis B virus X protein (HBx) is a main factor in the development of HBV-induced disease. HBx increases endoplasmic reticulum (ER) stress-dependent CXCL12 expression and mediates HBV-induced recruitment of immune cells into the liver via CXCL12 signaling (Cho et al., 2014). Furthermore, HBx is involved in the occurrence and development of HBV-related HCC through the CXCL12/CXCR4/β-catenin signaling axis (Wang C. et al., 2017). However, the inhibitory effect of AMD3100 on CXCR4 significantly suppressed CXCL12 signaling-mediated recruitment of immune cells in HBV liver, and significantly disrupted the effect of CXCL12 on the self-renewal capacity of HBx-expressing cancer stem-like cells (CSCs) in HBV-related HCC (Cho et al., 2014; Wang C. et al., 2017). During hepatitis E virus (HEV) infection, CXCR4 expression is increased in immune cells from the periphery in patients. Here, the overall profile of tissue-specific homing receptor CXCR4 expression on the surface of effector/memory peripheral lymphocytes suggests that these cells are targeted to homing specifically to the liver (TrehanPati et al., 2011). Altogether, these data indicate that CXCR4 and its ligand are essential for hepatitis and provide novel ideas for further diagnosis and treatment.

## The Protective Effect of CXCR4 Signaling Pathway in Acute Liver Injury and Regeneration

Acute liver injury is the manifestation of sudden hepatic injury and arises from a variety of causes, such as surgical resection, chemical exposure or ischemia/reperfusion (I/R) events. Liver regeneration is critical for acute restoration of liver mass after resection or injury (DeLeve, 2013). Liver regeneration after acute injury is always beneficial and has been intensively studied. Experimental models involving partial hepatectomy or chemical injury have revealed relevant cellular signaling pathways that are used to restore the liver to equivalent mass and function to those prior to injury (Kitto and Henderson, 2020; Michalopoulos and Bhushan, 2020). Notably, the CXCL12/CXCR4 axis has received widespread attention in these signaling pathways. The study found that CXCR4 conditional knock-out mice (i.e., CXCR4^*f/null*^ mice were crossed with MxCre mice to get MxCre-CXCR4^*f/null*^ mice; CXCR4 was conditionally deleted after induction of Cre expression by intraperitoneal injection of poly(I)-poly(C) (pIpC) in eight-week-old mice) are susceptible to severe liver injury, with increased mRNA expression of several markers related to liver injury and regeneration in the liver, suggesting that the CXCL12/CXCR4 signaling is essential for liver regeneration and prevention of liver disease progression (Tsuchiya et al., 2012).

Currently, mesenchymal stem cells (MSCs) from different sources are considered to have enormous potential in the treatment of acute liver injury (Deng et al., 2014; Xiu et al., 2020). These cells need to migrate to the injury sites to function, which may be regulated by the CXCL12/CXCR4 signaling axis. Indeed, the CXCL12/CXCR4 axis promotes the migration of MSCs to the injury sites to repair liver injury by differentiating into and fusing with hepatocytes (Hao et al., 2015). Moreover, targeted migration of MSCs modified with CXCR4 to acute failing liver improves liver regeneration (Ma et al., 2014). Similarly, migration and engraftment of MSCs overexpressing CXCR4 into liver grafts improves early liver regeneration of small-for-size liver grafts (Du et al., 2013). However, down-regulation of CXCL12 expression could suppress the directional migration of these MSCs to the injured liver (Lü et al., 2012). The migration of bone marrow mesenchymal stromal/stem cells (BMSCs) is also regulated by the CXCL12/CXCR4 signaling, which is involved in the recruitment of BMSCs to the injured liver, while AMD3100 or anti-CXCR4 antibody can block this migration (Xiao Ling et al., 2016). Interestingly, overexpression of CXCR4 in BMSCs can substantially promote their migration and result in even better therapeutic effects for acute liver injury. This may be attributed to the activation of PI3K/Akt signaling pathway in BMSCs that is downstream of CXCR4 (Xiu et al., 2020). The CXCL12/CXCR4 axis similarly regulates the migration of umbilical cord-derived mesenchymal stem cells (UC-MSCs) to the injured liver. Herein, the pretreatment of UC-MSCs by rapamycin increases CXCR4 expression, enhances the homing and migratory capacity of these cells through the CXCL12/CXCR4 axis and ameliorates liver I/R injury (Zheng et al., 2018). Furthermore, up-regulation of CXCR4 in UC-MSCs induced by serum from rats with acute liver failure also promotes the migration and homing ability of stem cells to the injured liver, which may ultimately be used to treat liver disease (Deng et al., 2014).

The CXCR4 signaling pathway has also been proven to promote the migration and directional distribution of other stem cells at the injury sites (Wu et al., 2015). Urine-derived stem cells (USCs) have strong self-renewal capacity and multi-directional differentiation potential. Hypoxia preconditioning promotes the proliferation, migration and cell fusion of USCs by inducing CXCR4 signaling, leading to liver tissue recovery following injury (Hu et al., 2021). Based on the mechanism of the CXCL12/CXCR4 axis, the systemically transplanted adipose-derived stem cells (ADSCs) home to the injured liver after transplantation can stimulate liver regeneration in hepatectomy and I/R injured model mice (Saito et al., 2014). In addition, bone marrow (BM) and hematopoietic stem cells also participate in liver regeneration and proliferation. CXCL12 is required for effective hematopoietic stem cells mobilization and homing to the liver after hepatectomy (Lehwald et al., 2014). Specifically, hematopoietic stem cells are released from the BM into the peripheral blood, and matrix metalloproteinase 9 (MMP9) contributes to the mobilization of BM cells in the injured liver by up-regulating the expression of CXCR4 on BM cells and attracting BM cells along their CXCL12 gradient (Kawai et al., 2012). Moreover, up-regulation of CXCL12 expression also increases recruitment and mobilization of CXCR7^+^ BM progenitors of LSECs in the liver and promotes liver regeneration (DeLeve et al., 2016). In summary, accumulating evidence indicates that the CXCR4 signaling pathway plays a vital role in the pathophysiology of liver injury and regeneration, and strategies targeting this pathway may therefore be of therapeutic potential.

## CXCR4 and Its Ligand in Liver Fibrosis

Liver fibrosis is the result of a sustained wound-healing response subsequent to chronic liver injury and aims to restore liver integrity after injury caused by different causes (Kamdem et al., 2018). If left untreated, advanced liver fibrosis can lead to cirrhosis, portal hypertension, and eventually HCC and liver failure (Marra and Tacke, 2014; Wang S. et al., 2017). Currently, academic opinion holds that a variety of cells, mainly HSCs, play a vital role in the pathophysiology of liver fibrosis (Higashi et al., 2017). In recent years, with the in-depth study of the mechanism of liver fibrosis, it has been found that CXCR4 and its ligand play a critical role in the pathogenesis of liver fibrosis via the activation and recruitment of various cells (Chen et al., 2014; Zhang et al., 2015). Here, the expression levels of CXCL12 and CXCR4 are significantly elevated in liver fibrosis and cirrhosis (Wald et al., 2004; Saiman et al., 2015; Xiang et al., 2017; Chalin et al., 2019). In response to CXCL12, cells (such as HSCs) expressing CXCR4 can participate in fibrosis and cirrhosis through migration and activation. HSCs are the cellular source of most of the extracellular matrix (ECM), and their activation and migration are the central link of liver fibrosis (Qin L. et al., 2018). Indeed, HSCs express CXCR4 receptor *in vitro* and *in vivo*, CXCR4 activation by CXCL12 directly promotes HSCs differentiation, proliferation and activation through the MAPK, ERK1/2 and PI3K/Akt pathways, which has a fibrotic effect (Hong et al., 2009; Chen et al., 2014). Moreover, CXCL12 acting on CXCR4 also promotes the contraction and activation of HSCs in a calcium-independent pathway (Saiman et al., 2013). Interestingly, studies have shown that CXCR4 expression can be induced in activated HSCs during the progression of liver fibrosis (Chow et al., 2016; Yang et al., 2019). The predominance of CXCR4 expression shift angiocrine response of LSECs, and reversely stimulate the proliferation of HSCs (Ding et al., 2013). Subsequently, the binding of CXCL12 to CXCR4 also induces HSCs proliferation and production of collagen I (Chow et al., 2016). In addition, in human immunodeficiency virus (HIV)/HCV co-infected livers, the HIV-1 × 4-envelope protein gp120 promotes the phosphorylation of ERK1/2 by interacting with CXCR4 on activated HSCs and has a pro-fibrogenic effects (Zheng et al., 2012). CXCR4 HIV also regulates the progression of liver fibrosis by inducing reactive oxygen species (ROS) production in HSCs and further promoting the expression of fibrogenesis-related genes (Lin et al., 2011). Given the critical role of HSCs activation in the progression of liver fibrosis, these studies suggest that specific targeting of CXCR4 and its ligand may be beneficial in liver fibrosis.

Currently, several studies have dissected the role of CXCR4 signaling pathway in HSCs and explored therapeutic interventions targeting this pathway in liver fibrosis. *In vitro* and *in vivo* studies have found that the inhibition of the CXCL12/CXCR4 biological axis in liver fibrosis can protect against the activation and migration of HSCs, and thus attenuates liver fibrosis (Liu et al., 2016; Qin L. et al., 2018; Sung et al., 2018; Ullah et al., 2019). Therefore, specifically targeting CXCR4 for the treatment of liver fibrosis has become a focus of research. Here, vascular endothelial growth factor (VEGF) siRNAs and CXCR4 antagonist AMD3100 encapsulated in nanoparticles (NPs) targeting CXCR4 can be delivered to fibrotic liver. Upon entry into the liver, VEGF siRNAs decrease VEGF expression, inhibit angiogenesis and normalize the distorted vessels in the fibrotic livers in the carbon tetrachloride (CCl4)-induced mouse model; AMD3100, as a targeting moiety, suppresses the progression of fibrosis by inhibiting the proliferation and activation of HSCs (Liu et al., 2016). Similarly, combined delivery of MEK inhibitor and sorafenib to the liver via CXCR4-targeted NPs prevents activation of ERK in activated HSCs and also has anti-fibrotic effects in the CCl4-induced mouse model (Sung et al., 2018). Furthermore, co-encapsulation of AMD3100 and pirfenidone into CXCR4-targeted combination liposomes for CXCR4 targeting displayed aggressive apoptosis in TGFβ-induced activated HSCs and significantly reduced α-SMA, suggesting a propensity to fibrosis regression (Ullah et al., 2019). But, surprisingly, in the chronic CCl4 model of liver injury, treatment of mice with AMD3100 did not improve hepatic fibrosis, and even aggravated liver fibrosis and inflammation with a specific increase in intrahepatic neutrophils (Saiman et al., 2015). The reason for this contradiction may be related to the targeted delivery method, as well as targeting different cells.

During liver fibrosis, CXCR4 pathway appears to be important for recruiting different cells to the injured liver, which may partly explain contradictory results of this pathway in the process of fibrosis and repair. In a mouse model of CCl4-induced liver fibrosis, the CXCL12/CXCR4 pathway is a critical chemotactic axis regulating the migration of MSCs from the bone marrow to the fibrotic liver, and recruited MSCs play different roles, including aggravating liver fibrosis and attenuating liver injury (Chen et al., 2009; Liu Y. et al., 2015). Notably, corticosterone can inhibit the recruitment and migration of MSCs via down-regulating CXCR4 and CXCR7 expression in MSCs (Zhang et al., 2015). In contrast, transplanted CXCR4-positive expanded endothelial progenitor cells (EPCs), induced by CXCL12 into the rat liver portal tracts, fibrous septa and hepatic sinusoids, effectively promote the remodeling of damaged tissues of liver fibrosis and suppress liver fibrogenesis (Nakamura et al., 2012). Taken together, CXCR4 and its ligand are functionally and mechanistically involved in the progression of liver fibrosis. However, simply blocking profibrotic CXCL12/CXCR4 axis is not sufficient to ameliorate liver fibrosis *in vivo*. Thus, it is necessary to adopt more cell types, combined with targeted delivery or specific strategies to modulate the CXCL12/CXCR4 signaling to target this pathway in liver fibrosis.

## CXCR4 Signaling Pathway in HCC

Hepatocellular carcinoma (HCC) is the most common primary malignant tumor of the liver with a high worldwide prevalence and poor prognosis (Hu et al., 2020). Metastasis is the main event leading to death in the vast majority of HCC patients (Ye et al., 2016; Yin et al., 2019). Recent studies have shown that the tumor microenvironment (TME) plays a crucial role in cancer metastasis and development (Ye et al., 2016; Chen x. et al., 2018). During the metastasis and development of HCC, there is neovascularization and the recruitment and migration of related cells in the TME (Shen et al., 2010; Katayama et al., 2019). Here, CXCR4 signaling is the major pathway involved in the above activities in the TME (Wang et al., 2016). Indeed, many studies have found that the expressions of CXCL12 and CXCR4 in peripheral blood of HCC patients are significantly increased, and CXCR4 expression is positively correlated with lymph node metastasis and poor outcome of HCC, affecting the prognosis of HCC patients (Xiang et al., 2009; Neve Polimeno et al., 2014; Toraih et al., 2016; Qin L. F. et al., 2018). Moreover, high levels of CXCL12 are also detected in malignant biopsies of HCC patients. CXCL12 plays a vital role in the recruitment of Treg cells into TME. Increased numbers of Treg cells were shown in peripheral blood as well as in the tumor tissue. In brief, CXCL12 secreted in the TME recruits CD4^+^CD25^+^ Treg cells to the tumor sites to contribute to the growth and prosperity of HCC (Shen et al., 2010). Notably, the secretory CXCL12 in turn regulates CXCR4 in endothelial cells, reticular fibers to modulate the TME and regulate neovascularization, which may contribute to the distant metastasis of HCC. Furthermore, increased CXCL12 concentration in the TME activates the CXCL12/CXCR4 axis and enhances the recruitment of EPCs to HCC, which also promotes tumor neovascularization (Wang et al., 2016; Tsai et al., 2020). Neovascularization is known to be one of the major characteristics of HCC. CXCR4 is selectively expressed on a fraction of tumor endothelial cells in HCC tissues, and high levels of CXCR4 tend to develop a sinusoidal vasculature in tumors, which can be used as a novel vascular marker for vessel sprouting in HCC tissues (Meng et al., 2017; Xu et al., 2017). Mechanistically, up-regulated CXCR4 expression on endothelial cells is mediated by the ERK pathway induced by inflammatory cytokines derived from tumor conditioned monocytes/macrophages (Meng et al., 2017). Activated CXCR4/ERK signaling pathway, in turn, promotes HCC metastasis through M2 macrophage polarization (Cai J. et al., 2020). Interestingly, CXCL12 enhances the expression of VE-cadherin, matrix metalloproteinase 2 (MMP2) and laminin5γ2 via CXCR4 in tumor cells (rather than endothelial cells), forming vascular-like channels that promote vascular mimicry (VM) formation and provide blood perfusion for HCC tissues (Yang et al., 2016). In addition, stimulation of the CXCL12/CXCR4 signaling contributes to organ colonization with blood circulating tumor cells in HCC (Tang et al., 2019). Increased CXCR4 expression on tumor cells also leads to invasion, migration and epithelial-mesenchymal transition (EMT) of HCC cells (Gao et al., 2018).

Therefore, the importance of CXCL12/CXCR4 signaling in HCC tumor cells can be foreseen. Indeed, CXCR4 and its ligand CXCL12 initiate cell migration and angiogenesis via activation of the MAPK/ERK and PI3K/Akt signaling pathways, thereby promoting HCC cells growth, proliferation, metastasis and invasion (Yang et al., 2019). Similarly, CXCR7 signaling can also promote angiogenesis as well as HCC cells growth, invasiveness and differentiation by activating MAPK/ERK and Akt signaling pathways (Lin et al., 2014; Xue et al., 2014; Chen et al., 2016). Interestingly, the CXCL12/CXCR4 signaling induces the expression of matrix metalloproteinase 10 (MMP10) in HCC cells through the ERK1/2 pathway, which contributes to angiogenesis, growth and dissemination of HCC, and in turn, HCC cells stably expressing MMP10 have increased CXCR4 expression and migration capacity. This reciprocal crosstalk between the CXCL12/CXCR4 signaling and MMP10 contributes to the metastasis and progression of HCC (García-Irigoyen et al., 2015). Moreover, CXCR4 stimulated by CXCL12 also triggers activation of heterotrimeric G proteins, which regulate the chemotaxis and migration of HCC cells. Specifically, CXCR4-induced signaling pathways, including Gαi, Annexin A2 and Rac, activate actin polymerization to migrate HCC cells (Li et al., 2019). Notably, CXCR4 serves as an important intracellular signal transducer, can relay matrix stiffness signals through ubiquitin domain-containing protein 1 (UBTD1)-mediated YAP signaling pathway to drive HCC progression (Yang et al., 2020). Recent studies have shown that CXCL12 improves cell invasion potential of HCC cells and CXCR4 overexpression is associated with aggressive characteristics and poor prognosis of HCC, while inhibition of CXCR4 activity via CXCR4 knockdown, AMD3100 or neutralizing antibody administration suppresses tumorigenesis of hepatoma cells *in vitro* and *in vivo* (Liu H. et al., 2015; Lu et al., 2015). Thus, the CXCL12/CXCR4 signaling has become an attractive target for the diagnosis and treatment of HCC.

The CXCR4 signaling pathway is receiving increasing attention because it is clear that targeting this pathway may be beneficial for HCC. Here, targeting CXCR4 by CRISPR/Cas9 in HCC cells can inhibit invasion, proliferation and migration, reverse EMT, increase chemosensitivity and decrease the malignancy of HCC *in vitro* and *in vivo* (Wang X. et al., 2017). In addition, the study found that emodin exerts anti-HCC effects by targeting and down-regulating CXCR4, which is related to its inhibition of CXCL12-induced invasion and migration in HCC cell lines (Man et al., 2013). Another study found that plumbagin restrains HCC angiogenesis, as well as HCC cell proliferation and invasion by inhibiting the CXCL12/CXCR4-CXCR7 axis (Zhong et al., 2019). Notably, inhibition of CXCR7 expression by transfection with CXCR7-short hairpin RNA (shRNA) could significantly inhibit HCC cells and tumor endothelial cells proliferation, invasion, migration and angiogenesis (Zheng et al., 2010; Zhao et al., 2017; Wu et al., 2018). Currently, sorafenib is a multitargeted tyrosine kinase inhibitor approved as a systemic anti-angiogenic agent for advanced HCC, but its clinical application is limited due to moderate therapeutic efficacy and high incidence of acquired resistance resulted from elevated levels of the CXCL12/CXCR4 signaling induced by prolonged sorafenib treatment (Zheng et al., 2019). Thus, targeting down-regulation of CXCR4 expression or intervention in the CXCL12/CXCR4 signaling pathway might overcome sorafenib evasion and resistance (Gao et al., 2015; Zheng et al., 2019). Indeed, formulation of sorafenib in CXCR4-targeted lipid-coated poly (lactic-coglycolic acid) (PLGA) NPs modified with AMD3100 efficiently delivers sorafenib into HCC and human umbilical vein endothelial cells to achieve cytotoxicity and anti-angiogenic effect *in vitro* and *in vivo*. This highlights the clinical potential of CXCR4-targeted NPs for delivering sorafenib and overcoming acquired drug resistance in HCC (Gao et al., 2015). Similarly, CXCR4-targeted PEGylated PLGA NPs could co-deliver sorafenib and metapristone (chemopreventive agent targeting SDF-1/CXCR4 axis) into HCC *in vitro* and *in vivo*, thereby enhancing cytotoxicity and synergistically suppressing HCC proliferation and resistance (Zheng et al., 2019). Furthermore, the co-delivery of CXCR4-targeted NPs with MEK inhibitor and sorafenib to HCC can increase the feasibility of dual RAF/MEK inhibition to overcome sorafenib treatment evasion in HCC (Chen et al., 2017). Notably, encapsulation of AMD3100 and anti-angiogenic substance VEGF siRNA into lipid-based NPs formulations targeting CXCR4, namely AMD-NPs, could effectively deliver VEGF siRNAs into HCC and down-regulate VEGF expression *in vitro* and *in vivo*. Inhibition of CXCR4 by AMD-NPs in combination with either conventional sorafenib treatment or VEGF siRNA induces synergistic anti-angiogenic effects and inhibits local and distant tumor growth in HCC (Liu J. Y. et al., 2015). Targeting CXCR4 with AMD3100 also prevents the polarization toward an immunosuppressive microenvironment after sorafenib treatment, suppresses HCC tumor growth, reduces metastasis and improves survival (Chen et al., 2015). Collectively, targeting the CXCR4 signaling pathway in combination with sorafenib may provide a promising approach for the safe and effective treatment of HCC.

## Concluding Remarks and Future Perspectives

There is strong evidence that CXCR4 and its ligand play a key role in multiple liver diseases such as hepatitis, liver injury and regeneration, liver fibrosis and cirrhosis, as well as in HCC. Specifically, CXCL12 finely regulates signal transduction by activating CXCR4 depending on the internal and external conditions of cells and the pathophysiology of the body, and then participates in the development of liver disease. In addition to its conventional role in mobilizing immune cells to the site of inflammation, the CXCR4 signaling pathway also mediates several cellular functions specific to liver disease, such as promoting the activation and proliferation of HSCs, and the migration and invasion of HCC cells (Table 1). Although not yet fully established, CXCR4 and its ligand would seem to have both beneficial and deleterious effects, depending on the type of cells they target. For instance, the CXCL12/CXCR4 axis induces migration of EPCs, which effectively promotes the remodeling of damaged tissues of liver fibrosis and suppress liver fibrogenesis; however, this axis also produces fibrotic effects by promoting the activation, proliferation and migration of HSCs. Thus, exploiting the pharmacological potential of targeting CXCR4 and its ligand in liver disease requires a better understanding of their divergent actions. The studies outlined in this review article support the view that modulation of CXCR4 and its ligand represents a viable approach in treating liver disease and that combination targeted therapy might become another safe and effective strategy for clinical liver disease treatment (summarized in Table 2). Importantly, although it seems very clear that CXCR4 and its ligand play crucial roles in the pathophysiology of several liver disease, it should be noted that the exact role of targeting different cells needs to be further studied before targeting the CXCL12/CXCR4 signaling to treat these diseases.

**TABLE 1:** Involvement of CXCR4 and its ligand in liver disease.

Disease	Cells	Function	References
Toxin-induced hepatitis	CD4^+^ T cells	LSECs promotes the transmigration of CXCR4^+^ total CD4^+^ T cells and accumulates in Concanavalin A-induced hepatitis by providing perivascularly expressed CXCL12	Lutter et al., 2015
	CD4^+^, CD8^+^ lymphocytes	In alcohol induced hepatitis, CXCR4 dependent migration of CD4^+^ and CD8^+^ lymphocytes into the liver increased significantly	Karim et al., 2013
Non-alcoholic steatohepatitis	CD4^+^ T cells	In NASH liver, CXCL12 and CXCR4 protein levels are significantly increased, and CD4^+^ T cells are hyperresponsive to CXCL12	Bigorgne et al., 2008; Li et al., 2020
	CD4^+^ T cells	CXCL12 promotes the recruitment of CD4^+^ T cells in NASH and is dependent on CXCR4	Boujedidi et al., 2014
Viral hepatitis	T cells	The CXCL12/CXCR4 pathway is involved in recruitment and retention of T cells in the liver during HCV and HBV infection	Wald et al., 2004
	CD8^+^ T cells	In peripheral blood cells from HBV patients treated with PEGylated IFN-α, CXCR4 is highly expressed in CD8^+^ T cells, which is inversely correlated with HBV DNA loads	Liu et al., 2012
	Immune cells	HBx mediates HBV induced recruitment of immune cells into the liver via CXCL12 signaling	Cho et al., 2014
	Immune cells	During HEV infection, CXCR4 expression is increased in peripheral immune cells of patients and is targeted to homing specifically to the liver	TrehanPati et al., 2011
Liver injury and regeneration	MSCs	The CXCL12/CXCR4 axis promotes the migration of MSCs to the injury sites to repair liver injury and improve liver regeneration	Du et al., 2013; Ma et al., 2014; Hao et al., 2015
	BMSCs	CXCL12/CXCR4 is involved in the recruitment of BMSCs to the injured liver and overexpression of CXCR4 in BMSCs can promote their migration and result in even better therapeutic effects for acute liver injury	Xiao Ling et al., 2016; Xiu et al., 2020
	UC-MSCs	Up-regulation of CXCR4 in UC-MSCs promotes the migration and homing ability of these cells to the injured liver	Deng et al., 2014; Zheng et al., 2018
	USCs	Hypoxia preconditioning promotes the proliferation and migration of USCs by inducing CXCR4 signaling, leading to liver tissue recovery following injury	Hu et al., 2021
	ADSCs	The CXCL12/CXCR4 axis regulates ADSCs transplantation into the injured liver, stimulates liver regeneration	Saito et al., 2014
	Hematopoietic stem cells	CXCL12 is required for effective hematopoietic stem cells mobilization and homing to the injured liver	Lehwald et al., 2014
Liver fibrosis	HSCs	CXCL12 activation of CXCR4 directly promotes HSCs differentiation, proliferation and activation via the MAPK, ERK1/2 and PI3K/Akt pathways with fibrotic effects	Hong et al., 2009; Chen et al., 2014
	HSCs	The CXCL12/CXCR4 axis promotes the contraction and activation of HSCs in a calcium-independent pathway, and induces HSCs proliferation and production of collagen I	Saiman et al., 2013; Chow et al., 2016
	HSCs	CXCR4 HIV promotes liver fibrosis by promoting the phosphorylation of ERK1/2 on activated HSCs and inducing ROS production in HSCs	Lin et al., 2011; Zheng et al., 2012
	MSCs	The CXCL12/CXCR4 axis regulates the migration of MSCs from the bone marrow to the fibrotic liver, aggravating liver fibrosis	Chen et al., 2009; Liu Y. et al., 2015
	EPCs	CXCL12 induced CXCR4-positive expanded EPCs transplanted into rat fibrotic liver effectively suppress liver fibrogenesis	Nakamura et al., 2012
HCC	CD4^+^CD25^+^ Treg cells	CXCL12 secreted in the TME recruits CD4^+^CD25^+^ Treg cells to the tumor sites to contribute to the growth of HCC	Shen et al., 2010
	Endothelial cells	The secretory CXCL12 modulates CXCR4 in endothelial cells to regulate neovascularization, which may contribute to the distant metastasis of HCC	Tsai et al., 2020
	EPCs	The CXCL12/CXCR4 axis enhances the recruitment of EPCs to HCC and promotes tumor neovascularization	Wang et al., 2016
	Endothelial cells	CXCR4 is selectively highly expressed on tumor endothelial cells and can be used as a novel vascular marker for vessel sprouting in HCC tissues	Meng et al., 2017; Xu et al., 2017
	HCC cells	The CXCL12/CXCR4 signaling forms vascular-like channels through HCC cells and contributes to organ colonization with blood circulating tumor cells in HCC	Yang et al., 2016; Tang et al., 2019
	HCC cells	Increased CXCR4 expression on tumor cells leads to migration, invasion and EMT of HCC cells	Gao et al., 2018
	HCC cells	The CXCR4/CXCL12 axis promotes HCC cells growth, proliferation, metastasis and invasion via activation of MAPK/ERK and PI3K/Akt signaling pathways	García-Irigoyen et al., 2015; Yang et al., 2019
	HCC cells	CXCR4 stimulated by CXCL12 triggers heterotrimeric G proteins activation, which regulate the migration and chemotaxis of HCC cells	Li et al., 2019

*ADSCs, adipose-derived stem cells; Akt, serine/threonine-protein kinase; BMSCs, bone marrow mesenchymal stromal stem cells; EMT, epithelial-mesenchymal transition; EPCs, endothelial progenitor cells; ERK1/2, extracellular signal-regulated kinases 1/2; HBV, hepatitis B virus; HCC, hepatocellular carcinoma; HCV, hepatitis C virus; HEV, hepatitis E virus; HIV, human immunodeficiency virus; HSCs, hepatic stellate cells; IFN-α, interferon-α; LSECs, liver sinusoidal endothelial cells; MAPK, mitogen-activated protein kinase; MSCs, mesenchymal stem cells; NASH, non-alcoholic steatohepatitis; PI3K, phosphoinositide-3-kinase; ROS, reactive oxygen species; TME, tumor microenvironment; UC-MSCs, umbilical cord-derived mesenchymal stem cells; USCs, urine-derived stem cells.*

**TABLE 2:** Summary of drug studies targeting CXCR4 and its ligand in animal models of liver disease.

Disease model	Drug	Main effects of pharmacological drug	References
NASH mice	AMD3100	Inhibits the chemotactic effect of CXCL12 to CD4^+^ T cells and reduces the number of CD4^+^ T cells that reach the liver	Boujedidi et al., 2014
HBV mice	AMD3100	Inhibition of CXCR4 significantly suppresses CXCL12 signaling mediated recruitment of immune cells in HBV liver	Cho et al., 2014
Liver injury rats	AMD3100	Blocking BMSCs migration to the injured liver	Xiao Ling et al., 2016
Liver fibrosis mice	AMD3100 VEGF siRNA	VEGF siRNAs and AMD3100 are encapsulated in NPs targeting CXCR4 and delivered to liver fibrosis, inhibiting angiogenesis and HSCs activation to suppress the progression of fibrosis	Liu et al., 2016
Liver fibrosis mice	Sorafenib MEK inhibitor	Combined delivery of MEK inhibitor and sorafenib to the liver via CXCR4-targeted NPs prevents ERK activation in activated HSC and has anti-fibrotic effects	Sung et al., 2018
Liver fibrosis mice	AMD3100	Failure to improve hepatic fibrosis and even aggravate liver inflammation and fibrosis with a specific increase in intrahepatic neutrophils	Saiman et al., 2015
HCC mice	Sorafenib AMD3100	Formulation of sorafenib in CXCR4-targeted PLGA NPs modified with AMD3100 efficiently delivers sorafenib into HCC and endothelial cells to achieve cytotoxicity and anti-angiogenic effect	Gao et al., 2015
HCC mice	Sorafenib Metapristone	CXCR4-targeted PEGylated PLGA NPs could co-deliver sorafenib and metapristone into HCC, thereby enhancing cytotoxicity and synergistically suppressing HCC proliferation and resistance	Zheng et al., 2019
HCC mice	Sorafenib MEK inhibitor	The co-delivery of CXCR4-targeted NPs with MEK inhibitor and sorafenib in HCC can increase the feasibility of dual RAF/MEK inhibition to overcome sorafenib treatment evasion in HCC	Chen et al., 2017
HCC mice	AMD3100 VEGF siRNA	Encapsulation of AMD3100 and VEGF siRNA into NPs targeting CXCR4 can effectively deliver VEGF siRNA to HCC and induce anti-angiogenic effects	Liu J. Y. et al., 2015

*AMD3100, CXCR4 antagonist; NPs, nanoparticles; PLGA, poly (lactic-coglycolic acid); VEGF, vascular endothelial growth factor.*

## Author Contributions

SW constructed the major theme and wrote the first draft of the manuscript. SG, YL, and XQ reviewed and revised the manuscript. JL and XL supported the work of the manuscript. All authors have read and approved the final manuscript before submission.

## Conflict of Interest

The authors declare that the research was conducted in the absence of any commercial or financial relationships that could be construed as a potential conflict of interest.

## Publisher’s Note

All claims expressed in this article are solely those of the authors and do not necessarily represent those of their affiliated organizations, or those of the publisher, the editors and the reviewers. Any product that may be evaluated in this article, or claim that may be made by its manufacturer, is not guaranteed or endorsed by the publisher.
